# Supplementary material for: Research on the impact of career advancement on the mental health of young workers
Source: PLoS One. 2025 Aug 13;20(8):e0328440. doi: 10.1371/journal.pone.0328440 (PMC12349127; doi:10.1371/journal.pone.0328440)
Supplement: Attachment — Submitted filename: Review Plos ONE April 2025.docx [file pone.0328440.s002.docx]

**Review Plos ONE**

**A study of the impactof career development on the health status of joung workers**

The topic of the manuscript is interesting, but several key methodological and other weaknesses need to be addressed before the manuscript can be published.

**Introduction**

The state of the literature is not adequately described, and there are hardly any references to international literature. The section on gender (From a gender perspective....) on page 2 lacks references to the literature, for example.

**Hypotheses**

Two hypotheses are put forward, but these are not the focus of the discussion. Instead, new questions and analyses are repeatedly presented, e.g. on heterogeneity analyses, differentiations between inside/outside the system, gender and income. These analyses come as a surprise and were not introduced earlier.

**Methods**

1. A methods section is missing, in which the data, the variables used and all methods employed are described. Some methods are described in the results section, some in footnotes. Please describe the data, all methods (including benchmark regression and matching methods) and all variables in the methods section. For example, it is not yet clear what is meant by ‘inside/outside the system’ or ‘whether there is a staff’.
2. The methods used do not allow the hypotheses to be tested. The hypotheses are questions that need to be answered using mediation analysis. For example, the question is whether limited career opportunities have a negative impact on subjective health via the negative consequences of working hours and health behaviour. In this sense, health behaviour, for example, represents a mediator of the relationship between career opportunities and health. However, mediation analyses are not calculated. Nevertheless, mechanisms are discussed without being investigated. Hypothesis 2 with relative deprivation is not answered.
3. Only cross-sectional data is available, which does not allow any conclusions to be drawn about the direction of the relationship. It may also be the case that poor health influences career development. This problem is not addressed; instead, the influence of career opportunities on health is always discussed. Correctly, no statements can be made about the direction of the effect; only the relationship between the two variables can be discussed.

**Results**

1. The tables have inadequate titles and labels for the rows and columns. Information is missing, e.g. about what is meant by ‘highly educated’. Please clarify this.
2. Many sensitivity analyses are performed due to the endogeneity problem. Although it is appropriate to address this problem, it makes it difficult to identify the common thread running through the results. It is suggested that these sensitivity analyses be moved to the appendix and that the results section focus on answering the hypotheses. Therefore, it is also recommended that the robustness test be moved to the appendix.
3. It is rather unusual to provide explanations for the findings in the results section. This is usually done in the subsequent discussion.
4. The question arises as to why career opportunities are referred to as a treatment effect. What is the treatment effect in this case? Different terms are also used for this variable career opportunities, such as promotion block, non-promotion, limited development or constrained workers. This is confusing; please use consistent terminology.
5. There may be an error in Table 2. Is it really the case that non-promotions have better health (higher values)?
6. As mentioned above, no mechanisms are tested, only bivariate correlations that are linked theoretically but not empirically. Mediation analysis, in particular causal mediation analysis, represents an appropriate empirical method for testing the mechanisms. This method should be used.
7. The discussion fails to place the findings in the context of previous research. No other studies that have come to similar or different conclusions are cited in the discussion. This should be done.
