# Supplementary material for: Research on the impact of career advancement on the mental health of young workers
Source: PLoS One. 2025 Aug 13;20(8):e0328440. doi: 10.1371/journal.pone.0328440 (PMC12349127; doi:10.1371/journal.pone.0328440)
Supplement: Attachment — Submitted filename: Response to Reviewers.docx [file pone.0328440.s004.docx]

Dear Editor and Reviewers，

Thank you for your letter and for the reviewers’ comments concerning our manuscript entitled “A study of the impact of career advancement on the health status of young workers?” (ID: PONE-D-25-09440). We are truly grateful for the valuable comments and suggestions provided by the reviewers. These comments are of great significance to us in perfecting the article and enhancing its practical and academic value. We highly value all comments from both reviewers, and the authors have thoroughly discussed and deliberated each point to refine the theoretical framework. We deeply appreciate this opportunity for revision and have consequently made substantial modifications to the original manuscript. While the core research question remains unchanged, we have narrowed our focus to a specific dimension of health status, which enhances the logical consistency and coherence of our research hypotheses. We sincerely hope these revisions meet with your approval.

The following ***italicized*** *parts* are the original text of your comments., which we have carefully addressed and revised accordingly. The parts in blue font are the explanations of the revisions, while the revised parts in the text are highlighted in red font.

We would like to express our gratitude for your hard work!

Kind regards,

All Authors

**Reviewer #1:**

***Overall Recommendations:***

***1. Thorough proofreading and language editing for improved clarity, readability, and consistency.***

***2. Better integration and synthesis of literature to clearly highlight research gaps and theoretical contributions.***

***3. Clearly link empirical findings back to theoretical frameworks throughout the manuscript.***

***4. Provide deeper interpretive insights, explain unexpected results explicitly, and clearly articulate practical implications and limitations.***

***5. Please kindly check journal guidelines to follow manuscript structure.***

***Specific Recommendations：***

***Abstract:***

1. ***Language is somewhat repetitive (e.g., repeated phrases like “development restriction”).***
2. ***The final sentence regarding mental health differences between low and high income groups is confusing and repetitive.***
3. ***The abstract should explicitly state why this research is important, briefly mention implications or recommendations.***

We sincerely appreciate the reviewers' attention to our abstract writing. We acknowledge that the original abstract did contain certain issues in its presentation. In response, we have completely rewritten the abstract. Firstly, we have narrowed our research focus specifically to workers' mental health and introduced the study background at the outset. Secondly, we have revised the presentation of research findings, eliminating the previous bullet-point format to avoid linguistic redundancy and enhance clarity. Finally, we have supplemented the discussion of the study's significance. We remain grateful for your guidance.

For your convenience in review, we present the revised abstract below (please refer to pages 1-2 of the manuscript for the complete version):

Mental health is an important component of overall health. Currently, the mental health issues among Chinese workers are becoming increasingly severe, especially under the backdrop of increased uncertainty in career advancement, where depression and anxiety issues among young workers are prominent. Based on the panel data from the China Family Panel Studies (CFPS) in 2018 and 2020, this paper constructs an OLS regression model to empirically analyze the impact of career advancement constraints on workers' mental health and its mechanism of action. The study finds that limited career advancement significantly increases workers' depression index. Pathways such as extended working hours, deteriorated health conditions, and distorted labor value concepts play a partial mediating role, thereby reducing mental health levels. Heterogeneity analysis shows that compared to female, low-income, and low-educated individuals, male, those with medium to high income, and higher education levels are more likely to experience a decline in mental health due to career advancement constraints. This study provides empirical evidence for understanding the impact of career advancement uncertainty on mental health and offers important insights for improving occupational mental health policies and enhancing worker welfare.——pages 1-2

***Introduction:***

1. ***Redundant phrasing ("three-piece work set" repeated unnecessarily).***
2. ***The paragraph structure is somewhat unclear, blending government policy context and empirical health statistics without sufficient transition.***
3. ***Missing explicit statement of the research gap that this paper will address in the Introduction itself.***

We sincerely appreciate the reviewers' attention to our introduction section. We have thoroughly revised this section to enhance its logical flow. In the restructured introduction, we first emphasize the significance of mental health by highlighting relevant policy initiatives implemented by the Chinese government, while presenting statistical evidence demonstrating the severity of mental health challenges among Chinese workers - thereby establishing the importance of our research focus. The second paragraph systematically examines how career advancement affects workers' mental health, with particular attention to the emerging "lying flat" phenomenon among Chinese youth, which forms the basis for our research motivation. The third paragraph succinctly outlines limitations in existing literature and specifies our study's contributions to address these gaps. We are deeply grateful for your comprehensive and constructive suggestions.

Given the substantial length of the revised introduction, we have opted not to reproduce its full content in this response letter for conciseness, and we sincerely appreciate the reviewers' understanding in this matter. The complete modifications can be found on pages 2-3 of the revised manuscript.

***Literature Review:***

1. ***Several awkwardly phrased sentences (e.g., overly complex and long sentences).***
2. ***Insufficient synthesis: the literature review currently feels like a series of unrelated studies rather than a coherent narrative.***
3. ***Little international comparison or broader theoretical context provided.***

We sincerely appreciate the reviewers' valuable comments regarding our literature review section. We acknowledge that the original version suffered from insufficient integration of relevant studies and lacked coherent logical progression. In response, we have completely restructured this section.

The revised literature review consists of two systematic parts: First, we comprehensively examine factors influencing workers' career advancement and employment quality, with particular focus on individual characteristics (gender, education level, age, etc.). These factors subsequently inform our selection of control variables and heterogeneity analysis. Second, we analyze determinants of workers' mental health at both macro and micro levels. The macro-level analysis explores socioeconomic impacts on mental health, particularly relevant to China's current high-risk labor market context. The micro-level investigation focuses on individual attributes (place of residence, age, education) and labor market performance (working hours, health status, job perceptions), which guide our choice of control variables and mechanism analysis. Throughout this revision, we have substantially expanded the theoretical foundation by incorporating numerous additional references.

We are profoundly grateful for your guidance, which has significantly enhanced the quality of our manuscript. Given the extensive length of the revised literature review, we have not reproduced it in full here, but the complete modifications can be found on pages 3-4 of the revised manuscript.

***Theoretical Analysis and Hypotheses:***

1. ***Theories are presented clearly, but could benefit from further critical reflection or acknowledgment of limitations.***
2. ***Some sentences are too long or convoluted, making them hard to follow.***

We sincerely appreciate the reviewers' insightful comments regarding our theoretical analysis and hypotheses section. Upon re-examining our original framework where the dependent variable encompassed both physical health (self-rated health) and mental health outcomes - despite statistically significant regression results - we identified potential logical overlaps in the mechanisms through which career advancement affects these distinct health dimensions (e.g., career advancement might influence mental health via physical health pathways). To enhance theoretical coherence, we have completely restructured this section to focus exclusively on mental health outcomes.

The revised theoretical framework proposes two distinct mechanisms:

**Mechanism 1**: Career advancement constraints compel workers to extend labor supply, which deteriorates physical health and consequently impairs mental health (supported by extant literature on health economics).

**Mechanism 2**: Career advancement constraints generate relative deprivation, distorting work values and eroding future confidence, ultimately undermining mental health (grounded in social psychology literature).

Below we excerpt the restructured theoretical analysis (complete details appear on pages 5-6 of the manuscript):

Career advancement typically promises higher future earnings and enhanced social status. However, the accompanying promotion pressure may induce workers to adopt unhealthy behaviors. According to the theory of extended compensation [28], workers often prolong their working hours to secure greater remuneration and obtain career advancement opportunities such as promotions or training. Yet workers face finite time resources, creating a trade-off between work and leisure. Prolonged working hours can lead to overexertion, which detrimentally affects physical health [29] and significantly compromises overall physiological well-being [30-31]. Notably, deteriorating physical health further undermines mental health [32-33]. Consequently, when confronted with career advancement constraints, workers are more likely to extend working hours in pursuit of higher earnings and development opportunities—a process that may trigger declines in physical health. Based on this reasoning, we propose the following hypothesis:

**Hypothesis 1:** Career advancement constraints will lead to prolonged working hours, which in turn deteriorate physical health and ultimately impair mental well-being.

The theory of relative deprivation posits that disparities between individuals can lead workers to experience cognitive gaps characterized by a “others-have-but-I-don't” perception [34]. On one hand, the sense of “inequality” stemming from relative deprivation generates frustration and stress, eroding confidence in the future and consequently impairing mental health [35-36]. On the other hand, when workers persistently face disadvantaged competitive positions, their upward mobility aspirations and emotional dissatisfaction [37] significantly diminish the efficacy derived from career advancement. This manifests as destructive voice behaviors - disparaging remarks about work and organizations that foster toxic work environments and negative values [38], further exacerbating adverse mental health effects [39].Career advancement inherently involves competition among workers. Therefore, when workers face career advancement constraints, the resulting anxiety and negative perceptions may not only distort positive work attitudes but also undermine future confidence, ultimately compromising mental health [40].

**Hypothesis 2:** Career advancement constraints are more likely to induce negative work values and loss of future confidence among workers, thereby adversely affecting their mental health.——pages 5-6

***Models and Variables:***

1. ***Model description contains minor language/grammatical issues (e.g., confusing notation).***
2. ***Justification for sample selection (ages 30-40) is clear but could be briefly re-emphasized.***
3. ***Explanation of why certain control variables are included could be more explicit.***

We are deeply grateful for the reviewers' constructive comments on our methodology section. In response to your suggestions and incorporating issues raised by another reviewer, we have thoroughly revised the "Model and Variables" subsection. The restructured section now systematically presents: (1) data sources, (2) variable selection (including justification for control variables), (3) model specification, and (4) descriptive statistics.

Notably, we have strengthened the rationale for focusing on workers aged 30-40 by explicitly referencing "Career Advancement Theory" as theoretical support. Furthermore, we now provide detailed classifications of all variable types with individual explanations, supplemented by a comprehensive coding table (Table 1). These modifications substantially enhance the methodological rigor and transparency of our study.

For your convenience, we excerpt key revisions below (complete details appear on pages 6-11 of the manuscript):

This study utilizes panel data from the 2018 and 2020 waves of the China Family Panel Studies (CFPS). Initiated in 2010, the CFPS covers tens of thousands of households across 25 provinces and municipalities, with biennial follow-up surveys. The data boasts extensive national representativeness, employing stratified and multi-stage sampling methods that capture approximately 95% of China's total population [41]. Moreover, the dataset comprehensively documents various dimensions of Chinese society, including economic and demographic characteristics, while providing detailed measures of core variables such as health status, health behaviors, and job promotion satisfaction - making it particularly suitable for this investigation. Given our focus on career advancement's impact on workers' mental health, we restrict our analysis to individual-level CFPS samples. To construct our working sample, we exclude students, agricultural workers, the unemployed, and those who have exited the labor force. Recognizing that substantial age variation may introduce heterogeneity in career advancement stages - potentially causing self-selection bias and endogeneity concerns - we further restrict our sample to workers aged 30-40. Career advancement theory [42] suggests this age cohort is typically in the establishment stage, where securing promotions and achieving upward mobility constitute primary professional objectives. Additionally, this narrower age range ensures respondents face relatively homogeneous work-life challenges during our study period, enhancing estimation accuracy. After eliminating observations with missing values, our final analytical sample comprises 2,316 individuals.——pages 6

**Table 1. Variable definitions and types.——page 8**

| **Variable name** | **Variable Definition** |
| --- | --- |
| *Dependent variable* |  |
| Depression index | Self-rated scores in the CESD-8 questionnaire:  Continuous variable |
| *Independent variable* |  |
| Career advancement | Respondent is not satisfied with promotion or has no opportunity for promotion: Yes=1  No=0 |
| *Control variables* |  |
| Gender | Male=1  Female=0 |
| Age | The year of the questionnaire minus the year of birth gives the age:  Continuous variable |
| Income | Monthly total income of the worker；Logarithmic processing |
| Education of worker | Illiterate=1 (i.e., no education)  Primary school = 2  Junior high school = 3  Senior high school = 4  Associate degree = 5  Bachelor’s degree = 6  Master’s degree = 7  Doctoral degree = 8 |
| Place of residence | Rural=1; Urban =0 |
| Marital status | No spouse (including divorced, widowed, and never married) = 0  Spouse ( including married living with a spouse, married but not temporarily living with a spouse, separated)= 1 |
| Number of children | The number of children currently owned by the worker：Continuous variable |
| Occupational nature | Whether the worker is a civil servant：Yes=1  No=0 |
| Family size | The total number of co-residing family members. |
| Per capita family income | The ratio of total family income to the total number of family members:  Continuous variable |
| Mechanism variables |  |
| Work hours | The number of hours worked per week by the worker:  Continuous variable |
| Health status change | Improved = 1, Same (no change) = 2, Worsened = 3 |
| Work values | Agreement with “Interpersonal relationships are more important than real talent”：Strongly disagree=1  Disagree=2  Neutral=3  Agree=4  Strongly agree=5 |
| Confidence in the future | Very low confidence=1  Low confidence=2  Neutral=3  High confidence=4  Very high confidence=5 |
| Instrumental variable |  |
| Career advancement of similar groups(Peer effect) | Calculate the average perception of career advancement among workers with the same province, age, gender, education level, and income level. |

***Statistical Description:***

1. ***Minor grammatical issues (e.g., "people with limited development accounted for…").***
2. ***Insufficient explanation of why certain descriptive statistics are important or meaningful.***

We sincerely appreciate the reviewers' attention to our descriptive statistics section. As rightly pointed out, certain inappropriate symbols were inadvertently included in our original descriptive statistics table due to oversight, and the data description lacked sufficient detail. In response, we have thoroughly revised this section, providing more comprehensive comparisons between the two sample groups. We are grateful for your valuable suggestions, which have significantly improved the rigor of our empirical presentation.

For your convenience, we excerpt key revisions below (complete details appear on page 11 of the manuscript):

Table 2 provides the statistical description for this study. The statistical description indicates that workers dissatisfied with their career advancement constitute 48.8% of the total sample. By comparing the means of the two groups of variables, it is evident that workers facing career advancement constraints have a higher depression index compared to those with better career advancement, suggesting a lower level of mental health, which is significant at the 1% level. Regarding individual characteristics, workers with career advancement constraints tend to be older, have lower educational levels, have more children, and are more likely to hold rural household registrations, with a lower probability of being public servants. This also reflects the current labor market's inherent age and educational level discrimination, and non-public sector workers report lower satisfaction with their career advancement, potentially due to poorer job stability within this group. There are no significant differences in gender and marital status between the two groups. In terms of family characteristics, workers dissatisfied with their career advancement tend to have larger family sizes and lower per capita income, which may be attributed to greater financial and support pressures within the family, leading to a negative perception of career advancement.——pages 11

**Table 2. Descriptive statistics.**

| **Variable** | **Full sample** | | **Career advancement=1** | | **Career advancement=0** | |  |
| --- | --- | --- | --- | --- | --- | --- | --- |
|  | **Mean** | **Std.Dev.** | **Mean** | **Std.Dev.** | **Mean** | **Std.Dev.** | **Difference** |
| Depression index | 13.39 | 3.48 | 13.86 | 3.66 | 12.95 | 3.25 | 0.90 *** |
| Gender | 0.57 | 0.50 | 0.58 | 0.49 | 0.57 | 0.50 | 0.02 |
| Age | 34.57 | 3.19 | 34.86 | 3.19 | 34.28 | 3.17 | 0.58 *** |
| Income | 10.65 | 0.94 | 10.47 | 0.96 | 10.82 | 0.88 | -0.34 *** |
| Education of worker | 5.24 | 1.51 | 4.86 | 1.48 | 5.60 | 1.44 | -0.73 *** |
| Place of residence | 0.59 | 0.49 | 0.66 | 0.47 | 0.52 | 0.50 | 0.14 *** |
| Marital status | 0.86 | 0.35 | 0.87 | 0.34 | 0.85 | 0.36 | 0.02 |
| Number of children | 1.42 | 0.75 | 1.47 | 0.77 | 1.37 | 0.74 | 0.10 *** |
| Occupational nature | 0.17 | 0.38 | 0.13 | 0.33 | 0.22 | 0.41 | -0.09 *** |
| Family size | 3.76 | 1.82 | 3.84 | 1.79 | 3.69 | 1.84 | 0.15 ** |
| Per capita family income | 48709.81 | 129978.9 | 38020.77 | 41090.88 | 58894.14 | 176588.42 | -20873.37*** |
| Obs. | 2316 | | 1130 | | 1186 | |  |
| Notes: The term “Difference” refers to the difference in the mean values between the samples whose career advancement is restricted and those whose career advancement is not restricted. We use t-test to examine whether the differences between the two groups of samples are statistically significant.  *p< 0.1  **p< 0.05  *** p< 0.01 | | | | | | | |

***Empirical Analysis and Results:***

1. ***Limited explanation of unexpected findings (e.g., negative relationship between high education and health).***
2. ***R² values are relatively low, and the manuscript does not discuss the implications of this sufficiently.***

We sincerely appreciate the reviewers' attention to our empirical analysis and results section. Given our refined focus on the dependent variable and modifications to control variable selection and coding during revision, the coefficient magnitudes of independent variables and statistical significance of controls have correspondingly changed. We have provided plausible interpretations for statistically significant control variables.

Regarding the concern about relatively low R² values, we conducted extensive literature review and offer the following explanations: As our study utilizes micro-level survey data, we incorporated time and region fixed effects to mitigate potential omitted variable bias arising from temporal and regional economic disparities that may influence workers' career advancement. The modest R² values resulting from this modeling approach reflect inherent methodological characteristics rather than model inadequacy:

First, fixed-effects models employ within transformation to eliminate time-invariant individual heterogeneity, focusing exclusively on how within-individual variations in explanatory variables affect the outcome. Consequently, when within-individual variations are limited, the model's explanatory power naturally decreases.

Second, individual fixed effects capture time-invariant characteristics that may substantially contribute to outcome variability. However, the fixed-effects framework treats these as unobserved heterogeneity, meaning that controlling for temporal and regional factors necessarily excludes their explanatory contribution, thereby reducing overall R².

Finally, our survey of comparable studies employing microdata with fixed-effects specifications [1][2] confirms that modest R² values are normative in such designs and do not indicate model invalidity. We hope this clarification addresses the reviewers' concerns satisfactorily.

Once again, we are deeply grateful for your invaluable suggestions.

Due to the extensive content of the empirical section, we will not display all of it here. (complete details appear on pages 11-18 of the manuscript)

**References:**

1. Jiang Z, Liu H, Deng J, Ye Y, Li D. Influence of intergenerational support on the mental health of older people in China. PLoS ONE (v1;2006). 2024;19: 22. doi:10.1371/journal.pone.0299986

2. Nie P, Otterbach S, Sousa-Poza A. Long Work Hours and Health in China. China Economic Review. 2015;33: 212–229. doi:10.1016/j.chieco.2015.02.004

***Endogeneity Issues:***

1. ***Explanation of PSM approach is excessively detailed in footnotes, disrupting readability.***
2. ***Presentation of kernel density plots could be improved visually.***
3. ***The reasoning behind choosing "******35-year-old threshold" is clear but could benefit from additional theoretical framing.***

We appreciate the reviewers' attention to our endogeneity issues. Following your suggestions, and incorporating the feedback from another reviewer, we have made certain revisions to address the endogeneity concerns. We have employed the instrumental variable method to tackle the reverse causality endogeneity issue and retained the propensity score matching (PSM) method to address the sample selection bias endogeneity issue. Due to space considerations, we have removed the kernel density plots in the revised manuscript and retained only the table calculating the average treatment effect on the treated (ATT) using the PSM method, namely Table 7.

In the original text, we also used the “35-year-old threshold” as an exogenous shock to examine the treatment effect, which was similarly aimed at resolving the sample selection bias issue. Although the results remain significant under the new model setup, we have decided to omit this analysis in the revised manuscript. This decision is partly because we have already addressed a similar issue using the PSM method, and partly because introducing the “35-year-old threshold” as an exogenous shock might lead to the introduction of new content, which could slightly deviate from the overall logic of this paper.

To ensure the rigor and standardization of our research, we have included the optimized PSM-related test figures in our response to your comments. We are grateful for the reviewers' understanding. In the revised manuscript, the section addressing endogeneity issues is on pages 14-16.

|  |  |  |
| --- | --- | --- |

***Mechanism Testing and Heterogeneity Analysis:***

1. ***Some results lack sufficient theoretical explanation (e.g., why working hours significantly impact mental health but not physical health).***
2. ***Slight repetition in explaining expected results and interpretations.***

We sincerely appreciate the reviewers' valuable comments regarding our mechanism analysis and heterogeneity examination. As rightly pointed out, the original mechanistic framework required substantial improvement. In response, we have thoroughly restructured the theoretical logic in the "Theoretical Analysis and Hypotheses" section. For the mechanism testing, we now employ a mediation effects approach under this refined framework, with detailed interpretations of the empirical results provided. We are deeply grateful for your expert guidance, which has significantly strengthened our study.

For the reviewers' convenience, we excerpt the key modifications below (complete details appear on pages 12-14 of the manuscript):

**Analysis of mechanisms**

The baseline results have already indicated that career advancement constraints significantly increase the depression index of workers, thereby exerting a negative impact on their mental health levels. Based on the theoretical analysis in the previous text, this paper will utilize mediation effects to analyze the relevant mechanisms.

**The impact of career advancement on health behaviors and health changes.** This paper uses working hours as a proxy variable for workers’ health behaviors; the longer the working hours, the more likely workers are to experience fatigue due to overwork, thereby affecting their mental health levels. This paper uses workers’ self-assessed ‘change in health status over the past year’ as a proxy variable for their physiological health changes. Column (1) of Table 4 below shows the direct impact of career advancement on mental health levels. From column (2), it is evident that career advancement constraints significantly increase workers’ working hours; for each one-unit increase in the possibility of career advancement constraints, workers’ working hours increase by 4.138, which is significant at the 1% level. From column (3), it is known that restricted career advancement extends working hours; for each one-unit increase in the possibility of career advancement constraints, workers’ depression index increases by 0.711, which is significant at the 1% level. From column (4), it is known that for each one-unit increase in the possibility of career advancement constraints, workers’ self-assessed health decreases by 0.092, which is significant at the 1% level. From column (5), it is known that career advancement constraints affect workers’ depression index by worsening their physiological health; for each one-unit increase in the possibility of career advancement constraints, workers’ depression index increases by 0.675, which is significant at the 1% level. In summary, it can be concluded that restricted career advancement may increase workers’ depression index by extending their working hours and worsening their health levels, thus validating Hypothesis 1.

**Table 4. Effect of mechanism variables on Work hours and Decline in health**

| **Variables** | **Depression index** | **Work hours** | **Depression index** | **Decline in health** | **Depression index** |
| --- | --- | --- | --- | --- | --- |
|  | （1） | （2） | （3） | （4） | （5） |
| Career advancement | 0.782*** | 4.138*** | 0.711*** | -0.092*** | 0.675*** |
|  | (0.148) | (0.625) | (0.151) | (0.023) | (0.147) |
| Work hours |  |  | 0.018*** |  |  |
|  |  |  | (0.005) |  |  |
| Decline in health |  |  |  |  | -1.159*** |
|  |  |  |  |  | (0.146) |
| Year FE | YES | YES | YES | YES | YES |
| Province FE | YES | YES | YES | YES | YES |
| Obs. | 2,316 | 2,316 | 2,316 | 2,316 | 2,316 |
| R-squared | 0.079 | 0.228 | 0.084 | 0.026 | 0.108 |

Notes: In parentheses, standard errors clustered at the province level. Standard errors in parentheses.

*p< 0.1

**p< 0.05

*** p< 0.01

**The impact of career advancement on labor values and future confidence.** This paper uses the degree of agreement with the statement ‘interpersonal relationships are more important than hard work’ as a proxy variable for workers’ labor value concepts; the higher the agreement with this view, the more negative the workers’ labor values are perceived to be. The paper uses workers’ self-assessed ‘confidence in the future’ as a proxy variable for their evaluation of their own future confidence. Column (1) of Table 5 still represents the direct impact of career advancement on mental health levels. From column (2), it is evident that career advancement constraints can distort workers’ labor values, leading them to believe that maintaining good interpersonal relationships is more important than doing good work, and for each one-unit increase in the possibility of career advancement constraints, the degree of agreement with this view increases by 0.188, which is significant at the 1% level. From column (3), it is known that restricted career advancement can increase the perception of this view; for each one-unit increase in the possibility of career advancement constraints, the depression index of workers increases by 0.699, which is significant at the 1% level. From column (4), it is known that for each one-unit increase in the possibility of career advancement constraints, workers’ confidence in the future decreases by 0.173, which is significant at the 1% level. From column (5), it is known that career advancement constraints can affect workers’ depression index by reducing their confidence in the future; for each one-unit increase in the possibility of career advancement constraints, workers’ depression index increases by 0.586, which is significant at the 1% level. In summary, it can be concluded that restricted career advancement may increase workers’ depression index by distorting their labor values and reducing their confidence in the future, thus validating Hypothesis 2.

**Table 5. Effect of mechanism variables on health level**

| **Variables** | **Depression index** | **work values** | **Depression index** | **Confidence in the future** | **Depression index** |
| --- | --- | --- | --- | --- | --- |
|  | （1） | （2） | （3） | （4） | （5） |
| Career advancement | 0.782*** | 0.188*** | 0.699*** | -0.173*** | 0.586*** |
|  | (0.148) | (0.041) | (0.148) | (0.037) | (0.141) |
| Work values |  |  | 0.432*** |  |  |
|  |  |  | (0.073) |  |  |
| Confidence in the future |  |  |  |  | -1.135*** |
|  |  |  |  |  | (0.093) |
| Year FE | YES | YES | YES | YES | YES |
| Province FE | YES | YES | YES | YES | YES |
| Obs. | 2,316 | 2,316 | 2,316 | 2,316 | 2,316 |
| R-squared | 0.079 | 0.051 | 0.092 | 0.047 | 0.151 |

Notes: In parentheses, standard errors clustered at the province level. Standard errors in parentheses.

*p< 0.1

**p<0.05

*** p< 0.01

***Robustness Tests:***

1. ***Limited explanation about why specific robustness checks were selected.***
2. ***Tables lack detailed interpretation, making them seem more routine than analytical.***

We are grateful to the reviewers for their attention to the robustness checks. As you rightly pointed out, our original manuscript did indeed lack detailed interpretation of the robustness test results and clear explanations for the selection criteria. We have supplemented these aspects accordingly. Thank you for your valuable suggestions.

Given the extensive content of the robustness checks, we will not present them in full here. Please refer to pages 16-17 of the revised manuscript for complete details.

***Conclusions:***

1. ***Recommendations are somewhat generic and could be more specific (e.g., suggesting how to practically enhance labor supervision).***
2. ***Limited explicit reflection on study limitations or directions for future research.***

We sincerely appreciate the reviewers' attention to the conclusion section of our manuscript. As you rightly pointed out, the original version suffered from overly generalized recommendations and lacked thorough discussion of study limitations. We have substantially revised these aspects by providing more concrete policy implications and a comprehensive analysis of methodological constraints. We are deeply grateful for your constructive suggestions, which have significantly strengthened the scholarly contribution of our work.

Given the extensive content of the revised conclusion and limitations section, we have opted not to reproduce it in full here, but the complete modifications can be found on pages 18-20 of the revised manuscript.

***References:***

1. ***Minor inconsistencies in formatting (e.g., missing years for some references).***
2. ***Some cited sources in the text appear to be missing or inconsistently listed in the bibliography.***

We are grateful to the reviewers for their attention to the references section. We acknowledge that the original manuscript indeed suffered from insufficient citations, particularly of international literature, as well as inconsistent citation formatting. In response, we have substantially expanded the reference list with additional scholarly sources, with special emphasis on incorporating more international publications. Furthermore, we have meticulously standardized all citation formats to ensure full compliance with academic conventions. We sincerely appreciate your valuable suggestions, which have significantly enhanced the academic rigor of our reference documentation.

Given the extensive content of the revised References section, we have opted not to reproduce it in full here, but the complete modifications can be found on page 21-25 of the revised manuscript.

**Reviewer #2:**

***A study of the impact of career development on the health status of young workers***

***The topic of the manuscript is interesting, but several key methodological and other weaknesses need to be addressed before the manuscript can be published.***

The authors wish to express their profound gratitude for the reviewers' recognition of our study's academic value, which serves as tremendous encouragement for our research team. We have meticulously examined each suggestion and implemented corresponding revisions point by point. These constructive comments have been instrumental in significantly enhancing the scholarly quality of our manuscript. We reiterate our sincerest appreciation for your invaluable guidance.

***Introduction***

***The state of the literature is not adequately described, and there are hardly any references to international literature. The section on gender (From a gender perspective....) on page 2 lacks references to the literature, for example.***

We sincerely appreciate the reviewers' valuable comments regarding the introduction section. As you rightly pointed out, the original version indeed suffered from insufficient literature citations in both the introduction and literature review sections. In response, we have completely restructured the paper and rewritten the introduction with substantially expanded references.

The revised introduction follows this logical framework: First, we clearly focus our research question on workers' mental health and establish the study's background at the outset. Second, we have modified the presentation of research findings by eliminating the previous bullet-point format to enhance narrative flow and avoid redundancy. Finally, we have strengthened the discussion of the study's academic and practical significance.

For the literature review, we have implemented a two-part structure: Part I systematically examines factors influencing workers' career advancement and employment quality, particularly individual characteristics (gender, education level, age, etc.), which subsequently inform our control variables and heterogeneity analysis. Part II investigates determinants of workers' mental health at both macro (socioeconomic development) and micro levels (individual attributes like place of residence, age, and education; labor market performance including working hours, health status, and job perceptions), reflecting China's current high-risk labor market context while guiding our selection of control and mechanism variables. Notably, we have expanded the reference list from 23 to 62 citations, significantly enhancing the study's scholarly foundation and credibility.

Given the substantial length of the revised introduction and literature review, we have not reproduced them in full here, but the complete modifications can be found on page 21-25 of the manuscript. We are deeply grateful for your constructive suggestions that have greatly improved our work.

***Hypotheses***

***Two hypotheses are put forward, but these are not the focus of the discussion. Instead, new questions and analyses are repeatedly presented, e.g. on heterogeneity analyses, differentiations between inside/outside the system, gender and income. These analyses come as a surprise and were not introduced earlier.***

We are deeply grateful for the reviewers' insightful comments regarding our theoretical analysis and hypotheses section. As you appropriately noted, our original manuscript presented two hypotheses in the theoretical section but subsequently combined mechanism analysis and heterogeneity testing, which inadvertently diminished the importance of validating theoretical hypotheses through mechanism analysis. To address this, we have thoroughly restructured the paper's organization:

First, we have established mechanism analysis as an independent subsection following the baseline regression (see pages 5-6 of the manuscript).

Second, while our original dependent variable encompassed both physical health (self-rated health) and mental health with statistically significant results in both regression models, upon re-examining our theoretical framework, we recognized potential logical overlaps in the mechanisms through which career advancement affects these distinct health dimensions (e.g., career advancement might influence mental health via physical health pathways). To enhance theoretical coherence, we have rewritten this section to focus exclusively on mental health outcomes. We hope this refined theoretical framework meets with your approval.

Third, we have repositioned the heterogeneity analysis after robustness checks as further analysis. In the conclusions and discussion section, we have added detailed interpretations of the heterogeneity results, supported by relevant citations to strengthen the credibility of our findings. The grouping variables for heterogeneity analysis (gender, education level, and income) were selected based on key characteristics identified in our literature review as significant factors influencing either career advancement or mental health (see pages 14-18).

We sincerely appreciate your expert guidance, which has been invaluable in enhancing the methodological rigor of our study. Given the extensive nature of these revisions, we are unable to present all modifications here but have highlighted key changes for your consideration. Your understanding would be greatly appreciated.

**Analysis of heterogeneity（pages 17-18）**

**Heterogeneity in gender.** Career advancement paths may differ among workers of different genders, hence the paper conducts separate regressions for male and female workers. Columns (1) and (2) of Table 9 show that for male workers, a one-unit increase in the likelihood of career advancement constraints leads to a rise in the depression index of 1.23, which is significant at the 1% level, but this effect is not significant for female workers.

**Heterogeneity in income level.** Considering that workers with different income levels may perceive career advancement differently, the paper categorizes 'workers' income' into low, medium, and high income groups. The regression results indicate that career advancement constraints mainly increase the depression index of medium and high-income workers. For each one-unit increase in the likelihood of career advancement constraints, their depression index increases by 0.738 and 1.089, respectively, both significant at the 1% level. However, there is no significant impact on the mental health levels of low-income workers.

**Heterogeneity in education level.** Taking into account that workers with different educational levels may have different expectations for career advancement and may start at different career points, the paper conducts separate regressions for workers with different educational levels. The results show that career advancement constraints increase the depression index of workers with higher education levels. For each one-unit increase in the likelihood of career advancement constraints, their depression index rises by 0.855, but this effect is not significant for workers with lower educational levels.

**Table 9. Heterogeneity analysis**

| **Variables** | **Depression index** | | | | | | |
| --- | --- | --- | --- | --- | --- | --- | --- |
|  | （1） | （2） | （3） | （4） | （5） | （6） | （7） |
|  | **Male** | **Female** | **Low- income** | **Middle-income** | **High- income** | **Low-education** | **High-education** |
| Career advancement | 1.230*** | 0.114 | 0.474 | 0.738*** | 1.089*** | -0.599 | 0.855*** |
|  | (0.204) | (0.219) | (0.326) | (0.212) | (0.286) | (0.627) | (0.152) |
| Year FE | YES | YES | YES | YES | YES | YES | YES |
| Province FE | YES | YES | YES | YES | YES | YES | YES |
| Obs. | 1,330 | 988 | 540 | 1,093 | 685 | 214 | 2,104 |
| R-squared | 0.118 | 0.076 | 0.148 | 0.097 | 0.129 | 0.306 | 0.086 |
| Notes: In parentheses, standard errors are robust standard errors.  *p< 0.1  **p< 0.05  *** p< 0.01 | | | | | | | |

***Methods***

1. ***A methods section is missing, in which the data, the variables used and all methods employed are described. Some methods are described in the results section, some in footnotes. Please describe the data, all methods (including benchmark regression and matching methods) and all variables in the methods section. For example, it is not yet clear what is meant by ‘inside/outside the system’ or ‘whether there is a staff’.***

We sincerely appreciate the reviewers' attention to our data and methodology section. As correctly identified, the original variable definitions contained several ambiguous and non-standard elements. We have thoroughly addressed this issue by completely rewriting the Materials and methods, including: data sources, variable selection, model setting, and statistical statistics.

Regarding the specific query about "inside/outside the system" and "whether there is a staff," we have clarified this measure assesses whether respondents are civil servants - a crucial distinction in China's labor context where civil service positions typically offer greater job stability and benefits compared to other occupations, resulting in systematic differences in career trajectories. This operationalization now explicitly appears in our variable definitions table (Table 1).

We are profoundly grateful for these expert and constructive suggestions, which have significantly enhanced our study's methodological rigor. The complete revisions can be found on pages 7-8 of the manuscript, while key modifications are excerpted below for your convenience. Thank you for your understanding.

**Variable selection**

**Dependent variable.** The dependent variable in this study is workers' self-rated mental health status, measured as a continuous variable. The CFPS employed the 8-item Center for Epidemiologic Studies Depression Scale (CESD-8) in both 2018 and 2020 survey waves to assess individuals' subjective psychological states. Specifically, respondents were asked to rate their frequency of experiencing the following symptoms: (1) feeling depressed, (2) difficulty concentrating, (3) sleep disturbances, (4) feeling happy (reverse-coded), (5) feeling lonely, (6) feeling sad, and (7) perceiving life as overwhelming. Higher composite scores indicate more severe depressive symptoms and consequently poorer mental health status.

**Independent variables.** The independent variable in this study is workers' “career advancement status.” Career advancement theory [42] posits that the essence of career advancement lies in the formation of workers' self-concept. Workers' self-concept comprises both perceived self and ideal self [43], where the former refers to self-evaluation based on current circumstances, while the latter represents the desired state workers aspire to achieve. When the perceived self and ideal self cannot be reconciled, this significantly impacts career advancement [44]. Promotion, as a critical component of individual career advancement, influences workers' life planning through associated improvements in compensation and benefits [45]. Consequently, when workers express dissatisfaction with current promotion opportunities or face a lack of advancement prospects, the discrepancy between perceived self and ideal self indicates constrained career advancement. Based on this framework, the study employs “promotion satisfaction” as a proxy variable to measure the disparity between ideal self and actual self, serving as an indicator of career advancement status. Specifically, workers reporting being “very dissatisfied” or “dissatisfied”with promotions or facing “no promotion opportunities” are classified as experiencing career advancement constraints (coded as 1). Conversely, those reporting being “very satisfied” or “satisfied” are considered to have unconstrained career advancement (coded as 0), creating a dummy variable for analysis.

**Control variables.** This study controls for key individual and household characteristics that may influence career advancement. At the individual level, we include: gender, age, income, education level, place of residence, number of children, marital status, and whether the individual holds a civil service position [46-47]. These variables account for physiological characteristics, human capital endowments, and job characteristics that prior research identifies as significant determinants of career progression. For household characteristics, we incorporate per capita household income and household size to capture resource constraints and dependency burdens that may exacerbate worker stress [48]. The specific coding schemes for all control variables are detailed in Table 1.

**Instrumental variables.** Considering that workers' mental health levels can, in turn, affect their perception of career advancement, this may lead to an endogeneity issue of reverse causality. To address this, we will adopt the concept of “peer effects” to construct instrumental variables [49], that is, calculating the average perception of career advancement among workers with the same “province, age, gender, education level, and income level”, as an instrumental variable for individual workers' career advancement. On the one hand, these workers with similar characteristics are more likely to be homogeneous groups; the better the career advancement prospects of a group, the more likely it is that an individual worker, as a member of that group, will also have better career advancement prospects. On the other hand, the career advancement status of similar groups does not directly affect the mental health of individual workers; it only affects their mental health by influencing their possibilities for career advancement, thus making this instrumental variable reasonable.

**Mechanism variables.** The variables related to the mechanism test in this paper are as follows: workers' working hours, deterioration in health status, work values and workers' confidence in the future.

**Table 1. Variable definitions and types.**

| **Variable name** | **Variable Definition** |
| --- | --- |
| *Dependent variable* |  |
| Depression index | Self-rated scores in the CESD-8 questionnaire:  Continuous variable |
| *Independent variable* |  |
| Career advancement | Respondent is not satisfied with promotion or has no opportunity for promotion: Yes=1  No=0 |
| *Control variables* |  |
| Gender | Male=1  Female=0 |
| Age | The year of the questionnaire minus the year of birth gives the age:  Continuous variable |
| Income | Monthly total income of the worker；Logarithmic processing |
| Education of worker | Illiterate=1 (i.e., no education)  Primary school = 2  Junior high school = 3  Senior high school = 4  Associate degree = 5  Bachelor's degree = 6  Master's degree = 7  Doctoral degree = 8 |
| Place of residence | Rural=1; Urban =0 |
| Marital status | No spouse (including divorced, widowed, and never married) = 0  Spouse ( including married living with a spouse, married but not temporarily living with a spouse, separated)= 1 |
| Number of children | The number of children currently owned by the worker：Continuous variable |
| Occupational nature | Whether the worker is a civil servant：Yes=1  No=0 |
| Family size | The total number of co-residing family members. |
| Per capita family income | The ratio of total family income to the total number of family members:  Continuous variable |
| *Mechanism variables* |  |
| Work hours | The number of hours worked per week by the worker:  Continuous variable |
| Health status change | Improved = 1, Same (no change) = 2, Worsened = 3 |
| Work values | Agreement with “Interpersonal relationships are more important than real talent”：Strongly disagree=1  Disagree=2  Neutral=3  Agree=4  Strongly agree=5 |
| Confidence in the future | Very low confidence=1  Low confidence=2  Neutral=3  High confidence=4  Very high confidence=5 |
| *Instrumental variable* |  |
| Career advancement of similar groups(Peer effect) | Calculate the average perception of career advancement among workers with the same province, age, gender, education level, and income level. |

2. ***The methods used do not allow the hypotheses to be tested. The hypotheses are questions that need to be answered using mediation analysis. For example, the question is whether limited career opportunities have a negative impact on subjective health via the negative consequences of working hours and health behaviour. In this sense, health behaviour, for example, represents a mediator of the relationship between career opportunities and health. However, mediation analyses are not calculated. Nevertheless, mechanisms are discussed without being investigated. Hypothesis 2 with relative deprivation is not answered.***

We are deeply grateful for the reviewers' insightful comments regarding our mechanism analysis section. As previously mentioned, through restructuring the logical relationships between mechanism variables and revising our theoretical framework, we have refined our focus specifically on workers' mental health outcomes. This approach has significantly enhanced the clarity of our theoretical logic and strengthened the coherence of our research objectives.

Furthermore, in the revised mechanism analysis, we have implemented formal mediation analysis techniques. This established methodological approach substantially improves the credibility and rigor of our mechanism validation. We sincerely appreciate your invaluable suggestions, which have been instrumental in elevating the quality of our study.

For the reviewers' convenience, we have excerpted key modifications below. The complete revised section can be found on pages 12-14 of the manuscript.

**Analysis of mechanisms**

The baseline results have already indicated that career advancement constraints significantly increase the depression index of workers, thereby exerting a negative impact on their mental health levels. Based on the theoretical analysis in the previous text, this paper will utilize mediation effects to analyze the relevant mechanisms.

**The impact of career advancement on health behaviors and health changes.** This paper uses working hours as a proxy variable for workers’ health behaviors; the longer the working hours, the more likely workers are to experience fatigue due to overwork, thereby affecting their mental health levels. This paper uses workers’ self-assessed ‘change in health status over the past year’ as a proxy variable for their physiological health changes. Column (1) of Table 4 below shows the direct impact of career advancement on mental health levels. From column (2), it is evident that career advancement constraints significantly increase workers’ working hours; for each one-unit increase in the possibility of career advancement constraints, workers’ working hours increase by 4.138, which is significant at the 1% level. From column (3), it is known that restricted career advancement extends working hours; for each one-unit increase in the possibility of career advancement constraints, workers’ depression index increases by 0.711, which is significant at the 1% level. From column (4), it is known that for each one-unit increase in the possibility of career advancement constraints, workers’ self-assessed health decreases by 0.092, which is significant at the 1% level. From column (5), it is known that career advancement constraints affect workers’ depression index by worsening their physiological health; for each one-unit increase in the possibility of career advancement constraints, workers’ depression index increases by 0.675, which is significant at the 1% level. In summary, it can be concluded that restricted career advancement may increase workers’ depression index by extending their working hours and worsening their health levels, thus validating Hypothesis 1.

**Table 4. Effect of mechanism variables on Work hours and Decline in health**

| **Variables** | **Depression index** | **Work hours** | **Depression index** | **Decline in health** | **Depression index** |
| --- | --- | --- | --- | --- | --- |
|  | （1） | （2） | （3） | （4） | （5） |
| Career advancement | 0.782*** | 4.138*** | 0.711*** | -0.092*** | 0.675*** |
|  | (0.148) | (0.625) | (0.151) | (0.023) | (0.147) |
| Work hours |  |  | 0.018*** |  |  |
|  |  |  | (0.005) |  |  |
| Decline in health |  |  |  |  | -1.159*** |
|  |  |  |  |  | (0.146) |
| Year FE | YES | YES | YES | YES | YES |
| Province FE | YES | YES | YES | YES | YES |
| Obs. | 2,316 | 2,316 | 2,316 | 2,316 | 2,316 |
| R-squared | 0.079 | 0.228 | 0.084 | 0.026 | 0.108 |

Notes: In parentheses, standard errors clustered at the province level. Standard errors in parentheses.

*p< 0.1

**p< 0.05

*** p< 0.01

**The impact of career advancement on labor values and future confidence.** This paper uses the degree of agreement with the statement ‘interpersonal relationships are more important than hard work’ as a proxy variable for workers’ labor value concepts; the higher the agreement with this view, the more negative the workers’ labor values are perceived to be. The paper uses workers’ self-assessed ‘confidence in the future’ as a proxy variable for their evaluation of their own future confidence. Column (1) of Table 5 still represents the direct impact of career advancement on mental health levels. From column (2), it is evident that career advancement constraints can distort workers’ labor values, leading them to believe that maintaining good interpersonal relationships is more important than doing good work, and for each one-unit increase in the possibility of career advancement constraints, the degree of agreement with this view increases by 0.188, which is significant at the 1% level. From column (3), it is known that restricted career advancement can increase the perception of this view; for each one-unit increase in the possibility of career advancement constraints, the depression index of workers increases by 0.699, which is significant at the 1% level. From column (4), it is known that for each one-unit increase in the possibility of career advancement constraints, workers’ confidence in the future decreases by 0.173, which is significant at the 1% level. From column (5), it is known that career advancement constraints can affect workers’ depression index by reducing their confidence in the future; for each one-unit increase in the possibility of career advancement constraints, workers’ depression index increases by 0.586, which is significant at the 1% level. In summary, it can be concluded that restricted career advancement may increase workers’ depression index by distorting their labor values and reducing their confidence in the future, thus validating Hypothesis 2.

**Table 5. Effect of mechanism variables on health level**

| **Variables** | **Depression index** | **work values** | **Depression index** | **Confidence in the future** | **Depression index** |
| --- | --- | --- | --- | --- | --- |
|  | （1） | （2） | （3） | （4） | （5） |
| Career advancement | 0.782*** | 0.188*** | 0.699*** | -0.173*** | 0.586*** |
|  | (0.148) | (0.041) | (0.148) | (0.037) | (0.141) |
| Work values |  |  | 0.432*** |  |  |
|  |  |  | (0.073) |  |  |
| Confidence in the future |  |  |  |  | -1.135*** |
|  |  |  |  |  | (0.093) |
| Year FE | YES | YES | YES | YES | YES |
| Province FE | YES | YES | YES | YES | YES |
| Obs. | 2,316 | 2,316 | 2,316 | 2,316 | 2,316 |
| R-squared | 0.079 | 0.051 | 0.092 | 0.047 | 0.151 |

Notes: In parentheses, standard errors clustered at the province level. Standard errors in parentheses.

*p< 0.1

**p<0.05

*** p< 0.01

3. ***Only cross-sectional data is available, which does not allow any conclusions to be drawn about the direction of the relationship. It may also be the case that poor health influences career development. This problem is not addressed; instead, the influence of career opportunities on health is always discussed. Correctly, no statements can be made about the direction of the effect; only the relationship between the two variables can be discussed.***

We sincerely appreciate the reviewers' attention to the conclusions of our study. We would like to provide the following clarifications: First, our research utilizes two-wave panel data (2018-2020) from the China Family Panel Studies (CFPS), which may not have been sufficiently emphasized in the original manuscript, potentially causing confusion. We deeply regret this oversight and have now explicitly highlighted this temporal framework in the revised manuscript, with additional discussion in the limitations section.

Second, regarding the endogeneity concern raised about reverse causality, we have taken this critical issue seriously. Following established econometric practices for addressing reverse causality, we have identified and incorporated a new instrumental variable based on the "peer effects" theoretical framework. The revised manuscript includes detailed justification for both the validity and relevance of this instrumental variable.

We hope these substantive revisions adequately address your concerns. Once again, we are profoundly grateful for your invaluable suggestions that have significantly strengthened our work. Given the extensive nature of these modifications, we have excerpted only key changes below for your consideration, with complete details available on page 14 of the revised manuscript. Your understanding would be greatly appreciated.

**Endogenous test**

**Instrumental variable.** Considering that workers' mental health levels can also affect their perception of career advancement, that is, when workers have a higher depression index, they may have a more negative evaluation of their own career advancement, which could lead to an endogeneity issue of reverse causality. To address this, we first employ the instrumental variable method to resolve the issue. As mentioned earlier, we calculate the average perception of career advancement among workers with the same “province, age, gender, education level, and income level” known as the “peer effect” as the instrumental variable for this study. Table 6 presents the regression results using the instrumental variable method. From column (1), it can be seen that the career advancement of homogeneous groups is positively correlated with individual workers' career advancement, meaning that the higher the likelihood of career advancement constraints within a homogeneous group, the more likely individual workers' career advancement is to be hindered. From the regression coefficient in column (2) of the model, it can be determined that for each one-unit increase in the likelihood of career advancement constraints, the depression index of workers increases by 0.791, which is significant at the 1% level. This does not significantly differ from the main regression results, thus we can consider our findings to be reliable.

**Table 6.** **Instrumental variable regression results.**

| **Variables** | （1） | （2） |
| --- | --- | --- |
|  | **First stage** | **Second stage** |
|  | Career advancement | Depression index |
| Career advancement |  | 0.791*** |
|  |  | （0.150） |
| Peer effect | 1.000*** |  |
|  | （0.001） |  |
| Year FE | YES | YES |
| Province FE | YES | YES |
| Obs. | 2,313 | 2,313 |
| F-value | 58395 | |
| Wald（P-value） | 27.90（0.000） | |
| Notes: Year and province fixed effects in all regression. In parentheses, standard errors are robust standard errors.  *p< 0.1  **p< 0.05  *** p< 0.01 | | |

***Results***

1. ***The tables have inadequate titles and labels for the rows and columns. Information is missing, e.g. about what is meant by*** ***‘highly educated’. Please clarify this.***

We are grateful to the reviewers for their valuable comments. We have carefully revised all table headings and supplemented the table contents with additional methodological details, including fixed effects specifications and relevant annotations. Regarding the definition of "highly educated" noted by the reviewers, we have thoroughly re-examined the data and modified this variable's operationalization. In the control variables section, we have replaced the ambiguous " highly educated " categorization with a standardized continuous measure of educational attainment based on years of schooling (see Table 1). For the heterogeneity analysis, we have defined "low educated " as workers who have not completed compulsory education (9 years), while all others are classified as " highly educated."

Given that these revisions affect multiple sections of the manuscript, we are unable to present all modifications here but have selected representative examples for review. The complete revised content can be found on pages 17-18 of the manuscript. We sincerely appreciate the reviewers' understanding and their constructive suggestions, which have significantly improved the precision and clarity of our study.

**Analysis of heterogeneity**

**Heterogeneity in education level.** Taking into account that workers with different educational levels may have different expectations for career advancement and may start at different career points, the paper conducts separate regressions for workers with different educational levels^[1]^. The results show that career advancement constraints increase the depression index of workers with higher education levels. For each one-unit increase in the likelihood of career advancement constraints, their depression index rises by 0.855, but this effect is not significant for workers with lower educational levels.

**Table 9. Heterogeneity analysis**

| **Variables** | **Depression index** | | | | | | |
| --- | --- | --- | --- | --- | --- | --- | --- |
|  | （1） | （2） | （3） | （4） | （5） | （6） | （7） |
|  | **Male** | **Female** | **Low- income** | **Middle-income** | **High- income** | **Low-education** | **High-education** |
| Career advancement | 1.240*** | 0.114 | 0.474 | 0.738*** | 1.089*** | -0.599 | 0.855*** |
|  | (0.204) | (0.219) | (0.326) | (0.212) | (0.286) | (0.627) | (0.152) |
| Year FE | YES | YES | YES | YES | YES | YES | YES |
| Province FE | YES | YES | YES | YES | YES | YES | YES |
| Obs. | 1,330 | 988 | 540 | 1,093 | 685 | 214 | 2,104 |
| R-squared | 0.118 | 0.076 | 0.148 | 0.097 | 0.129 | 0.306 | 0.086 |

2. ***Many sensitivity analyses are performed due to the endogeneity problem. Although it is appropriate to address this problem, it makes it difficult to identify the common thread running through the results. It is suggested that these sensitivity analyses be moved to the appendix and that the results section focus on answering the hypotheses. Therefore, it is also recommended that the robustness test be moved to the appendix.***

We are deeply grateful for the reviewers' invaluable suggestions, which inspired us to reconsider the manuscript's overall structure. Drawing upon the organizational frameworks of published articles in this journal [1][3], we have determined that the most logical presentation sequence for empirical results should be: baseline regressions, analysis of mechanisms, endogeneity and robustness test and heterogeneity analysis. This structure prioritizes the core analytical components (baseline results and mechanism validation) while positioning extended analyses (endogeneity, robustness, and heterogeneity) appropriately. All findings will be thoroughly discussed in the conclusion section with relevant citations to substantiate their academic rigor and reliability. We hope this restructuring meets with your approval.

Given the comprehensive nature of these organizational changes throughout the manuscript, we are unable to present all modifications here. We kindly refer the reviewers to pages 14-16 of the revised manuscript for complete details. Your expert guidance has been instrumental in enhancing the logical flow and scholarly presentation of our work, for which we express our sincere appreciation.

**References:**

1. Jiang Z, Liu H, Deng J, Ye Y, Li D. Influence of intergenerational support on the mental health of older people in China. PLoS ONE (v1;2006). 2024;19: 22. doi:10.1371/journal.pone.0299986

3. Zhao X, Shen L, Jiang Z. The impact of the digital economy on creative industries development: Empirical evidence based on the China. Rosak-Szyrocka J, editor. PLoS ONE. 2024;19: e0299232. doi:10.1371/journal.pone.0299232

3. ***It is rather unusual to provide explanations for the findings in the results section. This is usually done in the subsequent discussion.***

We sincerely appreciate the reviewers' constructive comments regarding our results presentation. In response, we have systematically reorganized the manuscript's structure by relocating all results interpretation to the "Conclusions and Discussion" section, where we now provide in-depth analysis of each finding supported by comprehensive literature citations that validate our conclusions. We are profoundly grateful for your expert guidance, which has significantly enhanced the scholarly rigor of our work.

For your convenience, we have excerpted representative revisions below, with the complete modified content available on pages 18-20 of the manuscript for your review.

**Conclusions and Discussion**

This paper utilizes panel data from the China Family Panel Studies (CFPS) for the years 2018 and 2020, employing OLS regression analysis to first examine the impact of workers' career advancement on their mental health levels. Secondly, by logically organizing existing literature and viewpoints, we have formulated relevant hypotheses regarding the underlying mechanisms and tested them using mediation effects. The paper also considers endogeneity issues within the model and employs instrumental variable methods and propensity score matching (PSM) to address these concerns. In addition, the paper conducts heterogeneity analyses for workers of different genders, income levels, and educational levels to explore how career advancement constraints among different groups may affect mental health. Finally, we also perform robustness checks on the research findings by adding control variables and expanding or excluding samples. Against the backdrop of increasingly fierce competition in the labor market, it is hoped that our study will raise societal awareness of the mental health levels of young workers, which has significant practical implications for improving the level of healthy human capital in China.

Firstly, the baseline regression results indicate that restrictions on workers' career advancement lead to a significant increase in their depression index. The essence of career advancement constraints is the uncertainty workers face about the future. Existing literature has shown that uncertainty and the negative beliefs it induces are significant factors in causing anxiety and depression [50, 51], which is consistent with our research findings. As the unemployment rate among young Chinese workers gradually increases and the uncertainty in the labor market grows, workers may find it difficult to achieve more satisfactory career advancement. Since early adulthood is the prime period for career advancement, constraints in career advancement imply a narrowing of upward mobility opportunities, which may increase the depression and anxiety of young workers, thereby reducing their mental health.

Secondly, the main mechanisms of the paper are twofold. On one hand, career advancement constraints can lead to extended working hours for workers and a deterioration in their health status, thereby increasing their depression index and reducing their mental health levels. Current research also indicates that extended working hours can cause symptoms of depression and anxiety among workers [50, 51], which aligns with our study's conclusions. On the other hand, the inequality caused by career advancement constraints can enhance workers' sense of relative deprivation, leading to discontented subjective emotions [52, 53]. This distorts workers' labor values, leading to more negative cognitions. Moreover, career advancement constraints imply a narrowing of upward mobility opportunities, which to some extent undermines workers' confidence in the future. Insufficient confidence can also lead to an increase in their depression index [56]. It is evident that longer working hours, deteriorated health conditions, and negative value concepts can also have a certain impact on workers' mental health.

Lastly, this paper conducts heterogeneity analysis based on individual characteristics of workers (gender, income, and education level). The results reveal that compared to women, career advancement constraints primarily increase the depression index of men, meaning that it more significantly affects the mental health of male workers. This may be due to the influence of male breadwinner culture, in which men in East Asian cultures are expected to bear more family financial responsibilities. For men, the stigma associated with setbacks in career advancement is stronger than for women [57], and career advancement constraints imply difficulty in improving future income levels, which may increase their psychological stress [58]. Current findings on the impact of income levels on depressive symptoms are relatively consistent, indicating that those with lower incomes have lower mental health levels [59][60], while the impact of education level on workers' mental health is inconclusive. On one hand, some studies suggest that a higher level of education can promote individual mental health [61], and on the other hand, some scholars find through correlation analysis that there is no significant relationship between the two [62]. Our study finds that compared to low-income and low-educated workers, career advancement constraints mainly elevate the depression index of workers with medium and high income levels and higher education levels. This may be because low-income, low-educated workers are often in more grassroots positions or are more likely to be in a state of career instability, whereas middle and high-income groups often have better development conditions at work, making it easier for them to meet the conditions or thresholds for career advancement. The abundance of career advancement opportunities makes them more sensitive to the state of their career advancement.

4. ***The question arises as to why career opportunities are referred to as a treatment effect. What is the treatment effect in this case? Different terms are also used for this variable career opportunities, such as promotion block, non-promotion, limited development or constrained workers. This is confusing; please use consistent terminology.***

We are deeply grateful for the reviewers' valuable suggestions. Regarding your inquiry about the treatment effects analysis, we would like to clarify that this approach was initially employed to address sample selection bias, operating on principles similar to the Heckman two-step method - requiring the independent variable to be dichotomous and necessitating an exogenous variable to mitigate endogeneity concerns. However, during our revision process, we identified superior methods for handling endogeneity (instrumental variable approach) while retaining propensity score matching (PSM) to address selection bias. Furthermore, considering that introducing the “35-year-old threshold” as an exogenous shock might introduce new content potentially deviating from our core analytical framework, we have consequently removed the treatment effects analysis from our manuscript. We sincerely appreciate your understanding of this methodological refinement.

Concerning terminology standardization issues, we have implemented comprehensive revisions throughout the manuscript. The term “career development” is now uniformly translated as “career advancement” while instances describing constrained career progression consistently use “career advancement constraints”. For operational measurement, we employ workers' promotion satisfaction as our proxy variable for career advancement status. We hope these improvements meet with your approval.

Given the extensive nature of these revisions, we are unable to present all modifications here but have included representative examples below. The complete revised content can be found on pages 15-16of the manuscript. Your understanding would be greatly appreciated.

5. ***There may be an error in Table 2. Is it really the case that non-promotions have better health (higher values)?***

We sincerely appreciate the reviewers' insightful inquiry regarding our dependent variable specification. In our original analysis, we employed both self-reported physical health and mental health measures as outcome variables, where workers without promotions exhibited higher numerical values on the mental health scale. We would like to clarify that the CFPS database utilizes the CES-D 8-item scale to assess depressive symptoms, with higher scores indicating more severe depression levels and consequently poorer mental health status. We deeply regret our initial failure to explicitly explain this inverse scoring convention, which understandably raised concerns. In the revised manuscript, we have provided comprehensive documentation of this measurement approach to rectify this oversight.

For the reviewers' convenience, we have included key explanatory additions below (complete details appear on pages 8-11 of the manuscript):

**Variable selection**

**Dependent variable.** The dependent variable in this study is workers' self-rated mental health status, measured as a continuous variable. The CFPS employed the 8-item Center for Epidemiologic Studies Depression Scale (CESD-8) in both 2018 and 2020 survey waves to assess individuals' subjective psychological states. Specifically, respondents were asked to rate their frequency of experiencing the following symptoms: (1) feeling depressed, (2) difficulty concentrating, (3) sleep disturbances, (4) feeling happy (reverse-coded), (5) feeling lonely, (6) feeling sad, and (7) perceiving life as overwhelming. Higher composite scores indicate more severe depressive symptoms and consequently poorer mental health status.

**Independent variables.** The independent variable in this study is workers' “career advancement status.” Career advancement theory [42] posits that the essence of career advancement lies in the formation of workers' self-concept. Workers' self-concept comprises both perceived self and ideal self [43], where the former refers to self-evaluation based on current circumstances, while the latter represents the desired state workers aspire to achieve. When the perceived self and ideal self cannot be reconciled, this significantly impacts career advancement [44]. Promotion, as a critical component of individual career advancement, influences workers' life planning through associated improvements in compensation and benefits [45]. Consequently, when workers express dissatisfaction with current promotion opportunities or face a lack of advancement prospects, the discrepancy between perceived self and ideal self indicates constrained career advancement. Based on this framework, the study employs “promotion satisfaction” as a proxy variable to measure the disparity between ideal self and actual self, serving as an indicator of career advancement status. Specifically, workers reporting being “very dissatisfied” or “dissatisfied”with promotions or facing “no promotion opportunities” are classified as experiencing career advancement constraints (coded as 1). Conversely, those reporting being “very satisfied” or “satisfied” are considered to have unconstrained career advancement (coded as 0), creating a dummy variable for analysis. ——pages 7

**Table 2. Descriptive statistics.（page 11）**

| **Variable** | **Full sample** | | **Career advancement=1** | | **Career advancement=0** | |  |
| --- | --- | --- | --- | --- | --- | --- | --- |
|  | **Mean** | **Std.Dev.** | **Mean** | **Std.Dev.** | **Mean** | **Std.Dev.** | **Difference** |
| Depression index | 13.39 | 3.48 | 13.86 | 3.66 | 12.95 | 3.25 | 0.90 *** |
| Gender | 0.57 | 0.50 | 0.58 | 0.49 | 0.57 | 0.50 | 0.02 |
| Age | 34.57 | 3.19 | 34.86 | 3.19 | 34.28 | 3.17 | 0.58 *** |
| Income | 10.65 | 0.94 | 10.47 | 0.96 | 10.82 | 0.88 | -0.34 *** |
| Education of worker | 5.24 | 1.51 | 4.86 | 1.48 | 5.60 | 1.44 | -0.73 *** |
| Place of residence | 0.59 | 0.49 | 0.66 | 0.47 | 0.52 | 0.50 | 0.14 *** |
| Marital status | 0.86 | 0.35 | 0.87 | 0.34 | 0.85 | 0.36 | 0.02 |
| Number of children | 1.42 | 0.75 | 1.47 | 0.77 | 1.37 | 0.74 | 0.10 *** |
| Occupational nature | 0.17 | 0.38 | 0.13 | 0.33 | 0.22 | 0.41 | -0.09 *** |
| Family size | 3.76 | 1.82 | 3.84 | 1.79 | 3.69 | 1.84 | 0.15 ** |
| Per capita family income | 48709.81 | 129978.9 | 38020.77 | 41090.88 | 58894.14 | 176588.42 | -20873.37*** |
| Obs. | 2316 | | 1130 | | 1186 | |  |

Notes: The term “Difference” refers to the difference in the mean values between the samples whose career advancement is restricted and those whose career advancement is not restricted. We use t-test to examine whether the differences between the two groups of samples are statistically significant.

*p< 0.1

**p< 0.05

*** p< 0.01

6. ***As mentioned above, no mechanisms are tested, only bivariate correlations that are linked theoretically but not empirically. Mediation analysis, in particular causal mediation analysis, represents an appropriate empirical method for testing the mechanisms. This method should be used.***

We sincerely appreciate the reviewers' attention to our mechanism analysis section. As correctly noted, we have implemented formal mediation analysis and strengthened our interpretation with supporting literature in the discussion section. Your expert suggestions have been invaluable in enhancing the methodological rigor of our study, for which we express our deepest gratitude.

The substantive improvements to our mechanism analysis, as previously outlined, can be found in detail on pages 12-14 of the revised manuscript.

7. ***The discussion fails to place the findings in the context of previous research. No other studies that have come to similar or different conclusions are cited in the discussion. This should be done.***

We sincerely appreciate the reviewers' valuable comments regarding our discussion section. As suggested, we have significantly strengthened the "Conclusions and Discussion" section by incorporating additional relevant literature to enhance the credibility of our findings. Furthermore, we have expanded the limitations subsection and added a dedicated "Future Research Directions" paragraph to better align with the journal's academic standards.

While we have previously highlighted key aspects of these improvements, the complete revisions can be found on pages 18-20 of the revised manuscript. We are deeply grateful for your constructive suggestions that have substantially improved the scholarly contribution of our work.

The above constitutes our comprehensive response to all reviewers' comments. We wish to reiterate our deepest gratitude to the reviewers for their invaluable insights and constructive suggestions.

The Author Team

1. The paper also classifies education levels based on whether workers have completed the compulsory education as stipulated by law (junior high school). Workers who have completed only compulsory education or less are considered to have a low level of education, while those with education beyond the compulsory level are regarded as having a higher level of education.
