# Supplementary material for: Research on the impact of career advancement on the mental health of young workers
Source: PLoS One. 2025 Aug 13;20(8):e0328440. doi: 10.1371/journal.pone.0328440 (PMC12349127; doi:10.1371/journal.pone.0328440)
Supplement: Attachment — Submitted filename: Response_to_Reviewers_auresp_2.docx [file pone.0328440.s005.docx]

Dear Editor and Reviewer，

We are extremely grateful for the opportunity to revise our manuscript, which is of great significance for improving its quality. We have taken your concerns very seriously and have made adjustments and modifications to the best of our ability, hoping to gain your approval.

The following ***italicized*** *parts* are the original text of your comments, which we have carefully addressed and revised accordingly. The parts in blue font are the explanations of the revisions, while the revised parts in the text are highlighted in red font.

We would like to express our gratitude for your hard work!

Kind regards,

All Authors

**Reviewer #3:**

***To me, this paper have simple conclusion which show promotion restriction will lead to bad mental health. this is logical. however, the complex statistics with lack of diagram have lead me to puzzle. I recommend reduce statistics with better diagrammatical presentation. otherwise, this is a complex paper with minimal interest to readers for my understanding.***

We are extremely grateful for the valuable comments provided by the reviewer. The authors have taken your concerns very seriously and have discussed this issue thoroughly with our collaborators at the earliest opportunity. As you pointed out, we have employed a substantial amount of statistical analysis to demonstrate the rationality and accuracy of our research, which may indeed be complex for some readers. In response to your suggestion to “reduce statistics with better diagrammatical presentation,” we have added illustrations to the PSM section to more intuitively show the differences before and after sample matching. Additionally, we have replaced the regression tables in the heterogeneity analysis section with figures, allowing for a more intuitive comparison of coefficient differences across different groups. We have also removed the robustness test section to mitigate the negative reading experience caused by excessive statistical analysis.

In addition to making the aforementioned changes, please allow the authors to explain the reasons for retaining most of the current statistical content:

1. Baseline Regression: This is the fundamental part of our investigation into the impact of career advancement on mental health. It directly demonstrates whether the conclusion of our research question holds true. Some of the control variables and fixed effects are included to meet the standard requirements of demographic econometric models, and thus, they need to be retained.

2. Mechanism Analysis: This section aims to explain why career advancement can affect workers' mental health, that is, to inform readers through which factors career advancement influences mental health. We have employed mediation effect analysis here, which is not only the most common and classic method in mechanism analysis but also a frequently used approach for mechanism testing in the PLOS ONE journal[1][2]. We will attach these references for you to verify our statements. Moreover, another reviewer explicitly requested the use of mediation effect methods, so we need to retain this section.

3. Endogeneity Issues: This is a crucial issue in our research field. We have successively employed instrumental variable and PSM methods. The instrumental variable is used to address the reverse causality problem. Specifically, both our dependent and independent variables are subjective. Career advancement constraints may lead to a decline in mental health. Conversely, poor mental health can also cause workers to perceive their career advancement negatively. If this reciprocal relationship is not addressed, it becomes difficult to determine whether the independent variable affects the dependent variable or vice versa, leading to biased estimation results. The instrumental variable is the best way to resolve this issue, and thus, it needs to be retained. PSM is used to address the problem of sample selection bias. It constructs a counterfactual framework to make the groups in the treatment and control groups more comparable, thereby more clearly identifying the impact of career advancement on workers' mental health and ensuring the accuracy of the estimation results. Regarding the complex statistical methods you mentioned, which are mainly in this section, we have considered whether to remove them. However, after referring to highly relevant articles published in PLOS ONE within the same research field as our study, we found that both methods are common and necessary. Therefore, we kindly ask the reviewer for understanding.

4. Robustness Test: This section tests the reliability of our conclusions through a series of criteria by changing the sample selection and model construction methods. Since this is not the core content of our paper, we are willing to remove it.

5. Heterogeneity Analysis: This section aims to explain why different groups show inconsistent changes in mental health after being affected by career advancement. It enriches our research and is a part that is almost always included in studies within this field. Considering the previous excessive statistical tables, we have replaced them with figures here to allow you to intuitively observe the changes in regression coefficients across different groups.

In summary, we have to retained these statistical analysis sections. We kindly ask for your understanding! Thank you for your understanding.

References：

1.  Wang C, Guo J, Xu W, Qin S. The impact of digital transformation on corporate green governance under carbon peaking and neutrality goals: Evidence from China. PLoS ONE (v1;2006). 2024;19: 21. doi:10.1371/journal.pone.0302432

2.  Zhao X, Shen L, Jiang Z. The impact of the digital economy on creative industries development: Empirical evidence based on the China. Rosak-Szyrocka J, editor. PLoS ONE. 2024;19: e0299232. doi:10.1371/journal.pone.0299232

To facilitate your review of our revisions, we present some of the modified contents as follows:

Finally, based on the kernel density plots of the treatment and control groups before and after matching, it is evident that the fit after matching is superior to that before matching. This indicates that the method effectively addresses the issue of sample selection bias. See Figure 1 below.（page 16）

|  |  |
| --- | --- |
| **Figure 1.**  **Kernel density matching plot for PSM** | |
|  | |

**Analysis of heterogeneity**（page 17）

**Heterogeneity in gender.** Career advancement paths may differ among workers of different genders, hence the paper conducts separate regressions for male and female workers. Figure2 shows that for male workers, a one-unit increase in the likelihood of career advancement constraints leads to a rise in the depression index of 1.23, which is significant at the 1% level, but this effect is not significant for female workers.

**Heterogeneity in income level.** Considering that workers with different income levels may perceive career advancement differently, the paper categorizes ‘workers’ income’ into low, medium, and high income groups. The regression results indicate that career advancement constraints mainly increase the depression index of medium and high-income workers. For each one-unit increase in the likelihood of career advancement constraints, their depression index increases by 0.738 and 1.089, respectively, both significant at the 1% level. However, there is no significant impact on the mental health levels of low-income workers.

**Heterogeneity in education level.** Taking into account that workers with different educational levels may have different expectations for career advancement and may start at different career points, the paper conducts separate regressions for workers with different educational levels. Figure 2 shows that career advancement constraints increase the depression index of workers with higher education levels. For each one-unit increase in the likelihood of career advancement constraints, their depression index rises by 0.855, but this effect is not significant for workers with lower educational levels.

**Figure 2. Heterogeneity analysis by group**

Finally, the authors would like to express their gratitude to the reviewer once again. For any issues that remain unresolved, we kindly ask for your understanding!

The Author Team
